# Dorsal horn neurons release extracellular ATP in a VNUT-dependent manner that underlies neuropathic pain

**DOI:** 10.1038/ncomms12529

**Published:** 2016-08-12

**Authors:** Takahiro Masuda, Yui Ozono, Satsuki Mikuriya, Yuta Kohro, Hidetoshi Tozaki-Saitoh, Ken Iwatsuki, Hisayuki Uneyama, Reiko Ichikawa, Michael W. Salter, Makoto Tsuda, Kazuhide Inoue

**Affiliations:** 1 Department of Molecular and System Pharmacology, Graduate School of Pharmaceutical Sciences, Kyushu University, 3-1-1 Maidashi, Higashi-ku, Fukuoka 812-8582, Japan; 2 Department of Life Innovation, Graduate School of Pharmaceutical Sciences, Kyushu University, 3-1-1 Maidashi, Higashi-ku, Fukuoka 812-8582, Japan; 3 Institute of Neuropathology, University of Freiburg, Neurozentrum Breisacherstraße 64, Freiburg 79106, Germany; 4 JST, CREST, 1-8-1 Inohana, Chuo-ku, Chiba 260-8670, Japan; 5 Institute for Innovation, Ajinomoto Co., Inc., Kawasaki 210-8681, Japan; 6 Department of Nutritional Science and Food Safety, Tokyo University of Agriculture, Tokyo 156-8502, Japan; 7 Program in Neurosciences & Mental Health, Hospital for Sick Children, Toronto, Ontario, Canada M5G 1X8; 8 Department of Physiology, University of Toronto, Toronto, Ontario, Canada M5S 1A8

## Abstract

Activation of purinergic receptors in the spinal cord by extracellular ATP is essential for neuropathic hypersensitivity after peripheral nerve injury (PNI). However, the cell type responsible for releasing ATP within the spinal cord after PNI is unknown. Here we show that PNI increases expression of vesicular nucleotide transporter (VNUT) in the spinal cord. Extracellular ATP content ([ATP]_e_) within the spinal cord was increased after PNI, and this increase was suppressed by exocytotic inhibitors. Mice lacking VNUT did not show PNI-induced increase in [ATP]_e_ and had attenuated hypersensitivity. These phenotypes were recapitulated in mice with specific deletion of VNUT in spinal dorsal horn (SDH) neurons, but not in mice lacking VNUT in primary sensory neurons, microglia or astrocytes. Conversely, ectopic VNUT expression in SDH neurons of VNUT-deficient mice restored PNI-induced increase in [ATP]_e_ and pain. Thus, VNUT is necessary for exocytotic ATP release from SDH neurons which contributes to neuropathic pain.

## Abstract (web summary)

Purinergic receptor activation by extracellular ATP in the dorsal horn contributes to neuropathic pain, but which cell types release ATP in this context is not known. The authors show in a mouse model of neuropathic pain that ATP is released by dorsal horn neurons, a process requiring the vesicular nucleotide transporter, VNUT.

## Graphical abstract

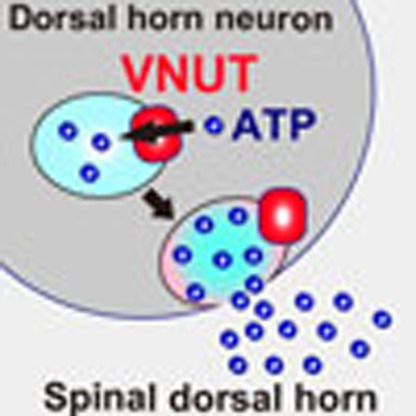

ATP is the well-known energy currency found in all living cells^1^. ATP also plays a distinct role in extracellular spaces, where it acts as a signalling molecule that controls cellular physiology, connectivity and dynamics through the activation of purinergic receptors^1^,^2^. Recently, extracellular ATP has emerged as a key player for a variety of diseases, including chronic inflammatory diseases^3^, and several neurological^4^,^5^ and psychiatric disorders^6^,^7^. Thus, characterizing the nature of ATP-mediated physiological phenomena is important for better understanding their aetiology, as well as identifying potential therapeutic targets of the diseases.

Neuropathic pain is one of the most debilitating chronic pain syndromes. It occurs concomitantly with neuronal damage as a consequence of multiple sclerosis, diabetes mellitus, cancer and traumatic injury^8^,^9^. Accumulating evidence has indicated the crucial roles of microglia, the immune-related glial cells of the central nervous system (CNS)^10^,^11^, in the spinal cord in the development of pain hypersensitivity following peripheral nerve injury (PNI). In response to PNI, spinal microglia transform into a reactive state through a sequence of cellular and molecular changes. These changes include morphological hypertrophy, proliferation and alteration in gene expression^12^,^13^ including genes encoding purinergic receptors^14^, such as ionotropic P2X4 receptor (P2X4R)^9^,^15^,^16^ and metabotropic P2Y_12_ receptor (P2Y_12_R)^17^ which are markedly upregulated after PNI^15^. In response to stimulation of these ATP receptors, microglia release several bioactive factors^18^,^19^, which cause abnormal neurotransmission in the dorsal horn nociceptive network^18^,^20^. These pathological alterations result in converting innocuous inputs to nociceptive signals^9^,^10^,^21^. The fact that the PNI-induced pain hypersensitivity is reversed rapidly by pharmacological blockade of spinal P2X4Rs^15^ or P2Y_12_Rs^17^ suggests that such pain behaviours require ongoing activation of these receptors by extracellular ATP in the spinal cord. These studies have led to two major questions: which type of cells release ATP, and what is the mechanism by which ATP is released from those cells, that causes pain hypersensitivity after nerve injury within the spinal cord^9^,^22^.

In the present study, we investigated the mechanisms and cell types that are involved in supplying extracellular ATP in the spinal cord. We also examined whether increased extracellular ATP release occurs within the spinal cord after PNI, and if it does, whether such ATP enhancement contributes to neuropathic pain. We identify vesicular nucleotide transporter (VNUT; also known as *Slc17a9*), a secretory vesicle protein responsible for the storage and release of ATP^23^,^24^, expressed in spinal dorsal horn (SDH) neurons as being crucial for exocytotic ATP release within the spinal cord and for neuropathic pain after PNI. Our findings may provide a novel target for treating neuropathic pain.

## Results

### Increase in spinal extracellular ATP after PNI requires VNUT

**Figure 1:**
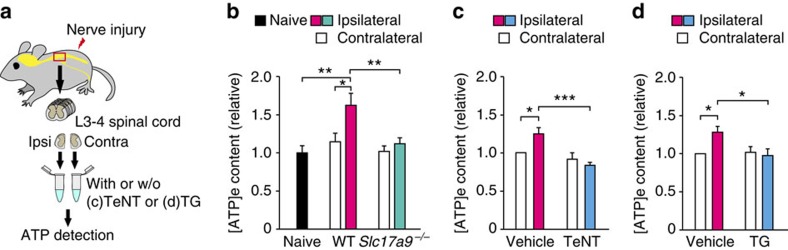
PNI-induced increase in extracellular ATP within the spinal cord is dependent on VNUT. (**a**) Schematic diagram of the experimental protocol for spinal ATP detection (Ipsi, ipsilateral; Contra, contralateral). (**b**) Measurement of extracellular ATP ([ATP]_e_) content in the ACSF media of ipsilateral and contralateral spinal cord slices taken from wild-type (WT) and VNUT-deficient (*Slc17a9*^−/−^) mice before (naive) and 7 days after PNI (naive: *n=*12, WT and *Slc17a9*^−/−^: *n=*16; *P<0.05, **P<0.01, one-way ANOVA with *post hoc* Tukey Multiple Comparison test). (**c**,**d**) Measurement of [ATP]_e_ content in the ACSF media of ipsilateral and contralateral spinal cord slices taken from WT mice with or without (w/o) (**c**) tetanus toxin (TeNT) or (**d**) thapsigargin (TG) 7 days after PNI (**c**: *n=*6, **d**: *n=*11; *P<0.05, ***P<0.001, one-way ANOVA with *post hoc* Tukey Multiple Comparison test). Values are means±s.e.m.

To date it is unknown if extracellular ATP ([ATP]_e_) level within the spinal cord is increased after PNI. Therefore, we first determined the [ATP]_e_ content within the spinal cord of wild-type (WT) mice subjected to PNI (Fig. 1a). Seven days after PNI, the concentration of [ATP]_e_ in the artificial cerebral spinal fluid (ACSF) incubated with spinal cord slices of the ipsilateral side was significantly higher than that of the contralateral side (*P<0.05*, Fig. 1b). A slight increase in [ATP]_e_ content was observed 3 days, but not 28 days, post-PNI (Supplementary Fig. 1). These results indicate that the level of [ATP]_e_ within the ipsilateral spinal cord is increased after PNI.

We next examined whether the increase in spinal [ATP]_e_ level involves VNUT. Notably, in the ipsilateral spinal cord of VNUT-deficient (*Slc17a9*^*−/−*^) mice^25^,^26^ the PNI-induced increase in [ATP]_e_ concentration was not observed (Fig. 1b). In contrast, there were no differences in [ATP]_e_ concentration in the contralateral sides between the two genotypes (Fig. 1b). VNUT-dependent ATP release is thought to be mediated via vesicular exocytosis^23^,^27^. We found that treatment with tetanus toxin, a neurotoxin that inhibits vesicular exocytosis^28^, markedly suppressed the PNI-induced increase in spinal [ATP]_e_ level (*P*<0.001, Fig. 1c). In addition, depletion of endoplasmic reticulum (ER) Ca^2+^ that is involved in exocytosis of neurotransmitter^29^ by thapsigargin, an inhibitor of the sarcoplasmic/endoplasmic ER Ca^2+^-ATPase, almost completely abolished spinal [ATP]_e_ increase (*P*<0.05, Fig. 1d), suggesting the importance of Ca^2+^ release from the ER for exocytosis of ATP-containing vesicles. Collectively, these results indicate that, in the ipsilateral spinal cord, PNI increases the amount of [ATP]_e_, which involves VNUT-dependent exocytosis.

### Reduction of PNI-induced allodynia in *Slc17a9* ^*−/−*^ mice

**Figure 2:**
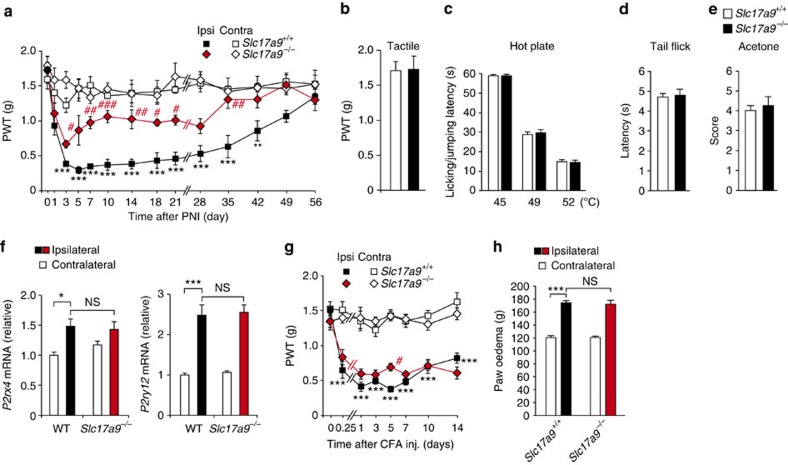
VNUT deficiency attenuates PNI-induced pain hypersensitivity without affecting acute pain sensation or inflammatory pain. (**a**) Paw withdrawal threshold (PWT) to tactile stimuli of *Slc17a9*^−/−^ mice and WT littermates (*Slc17a9*^+/+^) before and after PNI (*n=*5; ***P<0.001 versus the contralateral side of *Slc17a9*^+/+^ mice; ^#^*P<*0.05, ^##^*P<*0.01, ^###^*P<*0.001 versus the ipsilateral side of *Slc17a9*^+/+^ mice, two-way ANOVA with *post hoc* Bonferroni test). (**b**) PWT of *Slc17a9*^−/−^ and *Slc17a9*^+/+^ mice under normal conditions (*n=*5). (**c**) Hot-plate test, where values represent the latencies for animals to lick their hindpaws or jump (*n=*8). (**d**) Tail-flick test, where values represent the latencies to flick their tail from the heat source (*n=*8). (**e**) Acetone test, where values represent the pain scores after acetone application (*n=*8). (**f**) Real-time PCR analysis of mRNAs of *P2rx4* and *P2ry12* in the spinal cords of WT and *Slc17a9*^−/−^ mice 7 days after PNI. Values represent the relative ratio of mRNA (normalized to the value for 18S mRNA) to the contralateral side of WT mice (*n=*6; *P<0.05, ***P<0.001, one-way ANOVA with *post hoc* Tukey Multiple Comparison test). (**g**) PWT of *Slc17a9*^−/−^ and *Slc17a9*^+/+^ mice before and after intraplantar CFA injection (*n=*6; ***P<0.001 versus the contralateral side of *Slc17a9*^+/+^ mice; ^#^*P<*0.05 versus the ipsilateral side of *Slc17a9*^+/+^ mice, two-way ANOVA with *post hoc* Bonferroni test). (**h**) Paw size, as a measure of edema, in *Slc17a9*^−/−^ and *Slc17a9*^+/+^ mice 7 days after intraplantar CFA injection (*n=*6; ***P<0.001, one-way ANOVA with *post hoc* Tukey Multiple Comparison test). Values are means±s.e.m. NS, not significant.

To determine whether VNUT is involved in the pathogenesis of neuropathic pain, we used *Slc17a9*^*−/−*^ mice and assessed the severity of PNI-induced tactile allodynia—abnormal pain hypersensitivity evoked by innocuous stimuli. In WT littermate controls (*Slc17a9*^*+/+*^), the paw withdrawal threshold (PWT) to tactile stimuli in the ipsilateral hindpaw was markedly decreased following PNI (Fig. 2a), as previously reported^16^. *Slc17a9*^*−/−*^ mice also exhibited a reduction in PWT for the first 3 days after PNI, but failed to show a further decrease similar to WT littermates afterward (Fig. 2a), or rather showed a swift recovery from tactile allodynia (Fig. 2a). These results indicate that VNUT contributes to the production of neuropathic pain after PNI. Importantly, under normal conditions, *Slc17a9*^*−/−*^ mice showed normal mechanical sensitivity comparable to *Slc17a9*^*+/+*^ littermates (Fig. 2b). Furthermore, tail-flick, paw-flick and acetone tests revealed that acute physiological pain responses did not differ between the two genotypes (Fig. 2c–e), implying that the deficiency in VNUT does not cause a defect in general pain sensation. We also found that expression levels of P2X4R and P2Y_12_R in the SDH of *Slc17a9*^*−/−*^ mice were comparable to that of WT mice after PNI (Fig. 2f). Therefore, we concluded that VNUT-dependent enhancement of ATP release within the spinal cord is crucial for producing neuropathic pain.

We also assessed behavioural responses of *Slc17a9*^*−/−*^ mice against intraplantar injection of complete Freund's adjuvant (CFA), a well-established chronic inflammatory pain model. However, there were no differences in pain behaviours and the size of oedema in the paw between the two genotypes (Fig. 2g,h), suggesting that VNUT is not involved in chronic inflammatory pain. Taken together, these findings suggest that VNUT is the key player for the pathogenesis of chronic neuropathic pain, but not for acute physiological pain and local tissue inflammation-evoked pain.

### Increased VNUT expression in SDH is required for allodynia

**Figure 3:**
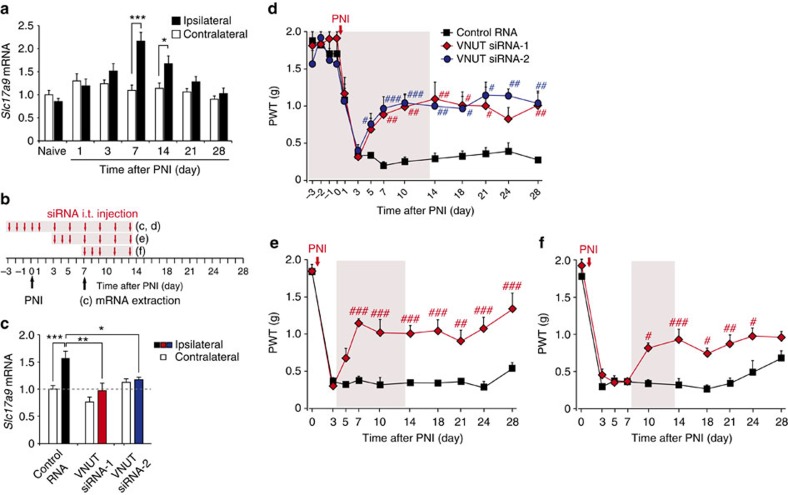
PNI-induced upregulation of VNUT in the spinal cord is required for the development of tactile allodynia. (**a**) Real-time PCR analysis of *Slc17a9* mRNA in total RNA extracted from spinal cord ipsilateral and contralateral to PNI before (naive) and after PNI. Values represent the relative ratio of *Slc17a9* mRNA (normalized to the value for 18S mRNA) to the contralateral side of naive mice (*n=*6; *P<0.05, ***P<0.001, two-way ANOVA with *post hoc* Bonferroni test). (**b**) Experimental schedule. (**c**) Real-time PCR analysis of *Slc17a9* mRNA in the spinal cords of mice treated with control or VNUT siRNAs (20 pmol per injection) 7 days after PNI. Values represent the relative ratio of *Slc17a9* mRNA (normalized to the value for 18S mRNA) to the contralateral side of mice with control RNA (control RNA: *n=*8, VNUT siRNA-1: *n=*5, VNUT siRNA-2: *n=*5; *P<0.05, **P<0.01, ***P<0.001, one-way ANOVA with *post hoc* Tukey Multiple Comparison test). (**d**–**f**) Reversal of PNI-induced tactile allodynia by intrathecal administration of VNUT siRNA (20 pmol per injection) in the ipsilateral side of WT mice (**d**, control RNA: *n=*5, VNUT siRNA-1: *n=*4, VNUT siRNA-2: *n=*5; **e**, *n=*5 per group; **f**, control RNA: *n=*4, VNUT siRNA-1: *n=*5; ^#^*P<*0.05, ^##^*P<*0.01, ^###^*P<*0.001 versus control RNA, two-way ANOVA with *post hoc* Bonferroni test). Values are means±s.e.m.

We next determined the temporal expression pattern of *Slc17a9* transcripts in the SDH following PNI by real-time PCR analysis. We found that expression of *Slc17a9* mRNA was markedly increased in the spinal cord ipsilateral to the injury, peaking at post-operative day 7 and persisting for >2 weeks (Fig. 3a). In contrast, there was no detectable change in the contralateral side (Fig. 3a). These results suggest that the upregulation of VNUT expression after PNI may be required for producing tactile allodynia. To test this concept, we intrathecally administered small interfering RNA (siRNA) targeting *Slc17a9* (Fig. 3b), which efficiently knocked down *Slc17a9* gene expression *in vitro* (Supplementary Fig. 2). Repeated administration of VNUT siRNA (siRNA-1) significantly suppressed the expression of spinal *Slc17a9* mRNA and ameliorated PNI-induced allodynic behaviour (Fig. 3c,d). Similar effects on gene expression and pain behaviour were obtained using an additional VNUT siRNA (siRNA-2) (Fig. 3c,d). Moreover, these siRNA treatments carried out during the maintenance phase also reversed established allodynia (Fig. 3e,f), the effects of which were continuously observed after the treatment (Fig. 3e,f). These results suggest that upregulated spinal VNUT plays a crucial role in the pathogenesis of neuropathic pain after PNI.

### Alterations of spinal microglia by PNI do not require VNUT

**Figure 4:**
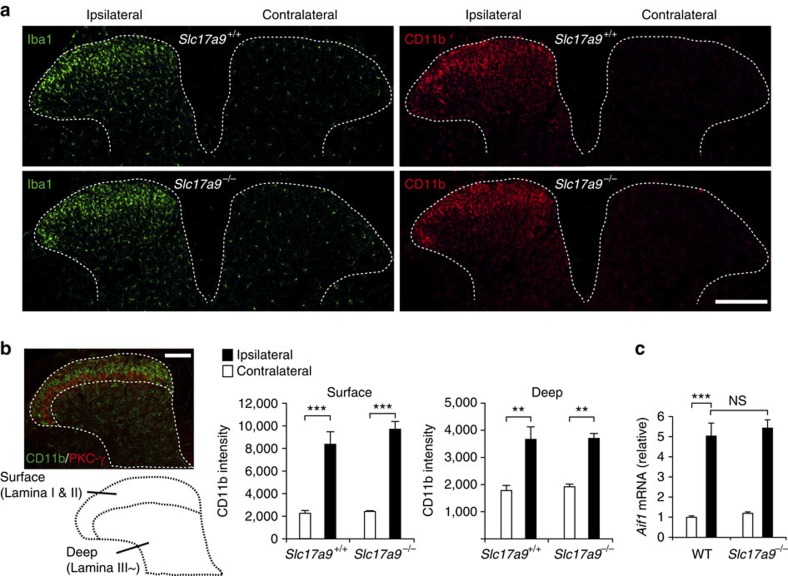
VNUT is not required for PNI-induced cellular alterations of spinal microglia. (**a**) Representative images showing immunofluorescence labelling of Iba1 (green) and CD11b (red) in the L4 spinal cord of *Slc17a9*^−/−^ and *Slc17a9*^+/+^ mice 7 days after PNI (scale bar, 200 μm). (**b**) The intensity of CD11b immunofluorescence was quantified for the surface (lateral side of PKC-γ signals) and deep (medial side of PKC-γ signals) dorsal horn region of *Slc17a9*^−/−^ and *Slc17a9*^+/+^ mice 7 days after PNI (*n=*4; **P<0.01, ***P<0.001, one-way ANOVA with *post hoc* Tukey Multiple Comparison test; scale bar, 100 μm). (**c**) Real-time PCR analysis of *Aif1* (Iba1) mRNA using total RNA extracted from the spinal cord of *Slc17a9*^−/−^ and WT mice 7 days after PNI. Values represent the relative ratio of *Aif1* mRNA (normalized to the value for 18S mRNA) to the contralateral side of WT mice (*n=*6; ***P<0.001, one-way ANOVA with *post hoc* Tukey Multiple Comparison test). Values are means±s.e.m. NS, not significant.

In response to PNI, robust proliferation and morphological changes in microglia occur in the spinal cord^13^, and are crucial for neuropathic pain^13^,^30^. Therefore, we tested the effect of VNUT deficiency on PNI-induced microglial cellular alterations. Notably, *Slc17a9*^*−/−*^ mice showed equivalent immunofluorescence patterns as WT mice in markers of microglia, ionized calcium-binding adapter molecule-1 (Iba1) and CD11b, in the SDH after PNI (Fig. 4a,b). Consistently, PNI increased the expression of *Aif1* transcripts that encode Iba1 in the spinal cord of *Slc17a9*^*−/−*^ mice to a similar extent as that observed in WT mice (Fig. 4c). These results indicate that VNUT is not required for PNI-induced cellular alterations of microglia in the spinal cord.

### VNUT in glial cells is not involved in PNI-induced allodynia

**Figure 5:**
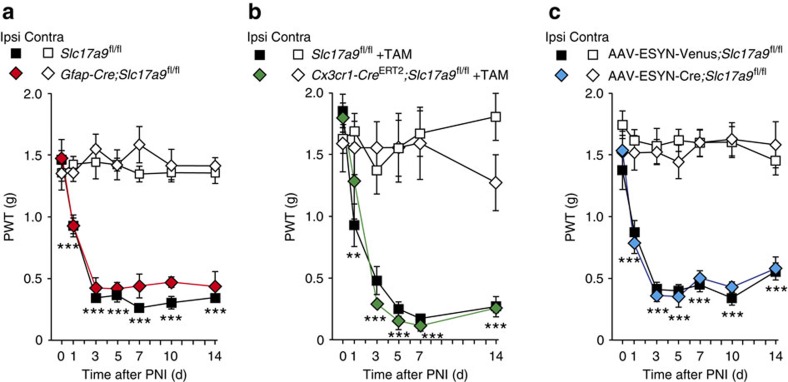
Astrocytes, microglia and primary sensory neurons are not involved in VNUT-dependent neuropathic pain after PNI. (**a**) PWT of astrocyte-specific VNUT conditional knockout (*Gfap-Cre*;*Slc17a9*^fl/fl^) mice and their *Slc17a9*^fl/fl^ littermate controls before and after PNI (*n=*6; ***P<0.001 versus Contra (*Slc17a9*^fl/fl^), two-way ANOVA with *post hoc* Bonferroni test). (**b**) PWT of microglia-specific VNUT conditional knockout (*Cx3cr1-Cre*^*ERT2*^;*Slc17a9*^fl/fl^) mice and their *Slc17a9*^fl/fl^ littermate controls before and after PNI. Both genotypes were injected with tamoxifen (TAM) 5 weeks before PNI. (*Slc17a9*^fl/fl^: *n=*6, *Cx3cr1-Cre*^*ERT2*^;*Slc17a9*^fl/fl^: *n=*5; **P<0.01, ***P<0.001 versus Contra (*Slc17a9*^fl/fl^ +TAM), two-way ANOVA with *post hoc* Bonferroni test). (**c**) PWT of DRG neuron-specific VNUT conditional knockout (AAV-ESYN-Cre;*Slc17a9*^fl/fl^) mice and control AAV-ESYN-Venus; *Slc17a9*^fl/fl^ mice before and after PNI (AAV-ESYN-Venus; *Slc17a9*^fl/fl^: *n=*8, AAV-ESYN-Cre;*Slc17a9*^fl/fl^: *n=*7; ***P<0.001 versus Contra (AAV-ESYN-Venus; *Slc17a9*^fl/fl^), two-way ANOVA with *post hoc* Bonferroni test). Values are means±s.e.m.

We sought to clarify which VNUT-expressing cell types are responsible for pain hypersensitivity. Having determined that siRNA-mediated knockdown of spinal VNUT expression suppressed pain behaviours of PNI-mice (Fig. 3), we reasoned that VNUT-expressing cells residing in the spinal cord may be crucial for tactile allodynia. Because one potential candidate for VNUT expression is astrocytes^7^,^31^,^32^, the most abundant CNS-resident glial cells, we crossed VNUT-floxed (*Slc17a9*^fl/fl^) mice with transgenic mice bearing a Cre recombinase under the control of a glial fibrillary acidic protein (GFAP) promoter (*Gfap-Cre* mice)^33^, and generated *Gfap-Cre*;*Slc17a9*^fl/fl^ mice in which VNUT is preferentially deleted in astrocytes. However, contrary to our expectations, *Gfap-Cre*;*Slc17a9*^fl/fl^ mice did not show any attenuation of pain behaviours after PNI, comparable to their *Slc17a9*^fl/fl^ littermate controls (Fig. 5a). These results indicate that astrocytic VNUT is not necessary for PNI-induced tactile allodynia.

Microglia also have a potential to express VNUT and release ATP^34^. To investigate the involvement of microglial VNUT in neuropathic pain, we crossed *Slc17a9*^fl/fl^ mice with mice possessing a tamoxifen-inducible Cre recombinase under the control of a *Cx3cr1* promoter (*Cx3cr1-Cre*^ERT2^ mice)^35^, and their 4–6 weeks-old offspring were treated with tamoxifen. After 5 weeks for which VNUT-deficient short-lived CX3CR1^+^ peripheral cells, but not long-lived microglia, are replaced by non-recombined cells continuously generated from CX3CR1^−^ progenitors within the bone marrow^35^, we subjected PNI to *Cx3cr1-Cre*^ERT2^;*Slc17a9*^fl/fl^ mice in which VNUT is preferentially deleted in microglia. We found that *Cx3cr1-Cre*^ERT2^;*Slc17a9*^fl/fl^ mice showed strong allodynic behaviour (Fig. 5b) and enhanced spinal [ATP]_e_ content (Supplementary Fig. 3a), indistinguishable from those of *Slc17a9*^fl/fl^ littermate controls (Fig. 5b and Supplementary Fig. 3a). In addition, *Cd11b-Cre*;*Slc17a9*^fl/fl^ mice, in which microglia do not express VNUT also exhibited comparable allodynic behaviours to their littermate *Slc17a9*^fl/fl^ controls (Supplementary Fig. 3b). These results suggest that microglial VNUT is not involved in neuropathic pain after PNI.

### VNUT in primary afferents is not required for allodynia

It has been assumed that ATP may be released from central terminals of primary sensory neurons in the SDH^36^,^37^,^38^. In addition, recent histological analysis^39^ showed that VNUT immunofluorescence is observed in dorsal root ganglion (DRG) neurons. Therefore, we investigated whether VNUT expressed in primary sensory neurons participate in producing neuropathic pain. To efficiently target DRG neurons, we performed a viral vector-mediated gene transduction^40^, in which neonatal mice were injected i.p. with an AAV2/9 (adeno-associated virus type 2 pseudotyped with AAV9 capsid) vector encoding the Cre recombinase under the control of neuron-specific promoter, human enhanced synapsin (ESYN) (AAV-ESYN-Cre). To verify Cre recombinase activity in DRG neurons, we injected AAV-ESYN-Cre into neonates of ROSA26 (R26)-tdTomato reporter mice, and found efficient Cre activity in almost all DRG neurons (96.1%: 937 of 975 total DRG neurons positive to Nissl staining) with less effect on SDH neurons (1.34%: 30 of 2,247 NeuN^+^ neurons) (Supplementary Fig. 4a,h,i). In addition, co-expression of tdTomato fluorescence with neuronal markers showed that 95.3% of NF200^+^ cells (304 of 319 cells), 95.0% of P2X3R^+^ cells (403 of 424 cells) and 96.4% of IB4^+^ cells (373 of 387 cells) were also tdTomato^+^ (Supplementary Fig. 4b–g). We then generated mice that had VNUT deleted in their DRG neurons by injecting the AAV-ESYN-Cre into *Slc17a9*^fl/fl^ neonatal mice (AAV-ESYN-Cre;*Slc17a9*^fl/fl^ mice), and performed behavioural analysis. Following PNI, AAV-ESYN-Cre;*Slc17a9*^fl/fl^ mice displayed a decrease in PWT that was indistinguishable from control *Slc17a9*^fl/fl^ mice that received AAV-ESYN-Venus (Fig. 5c). These results suggest that PNI-induced pain behaviour does not require VNUT in DRG neurons.

### VNUT in SDH neurons contributes to ATP release and pain

**Figure 6:**
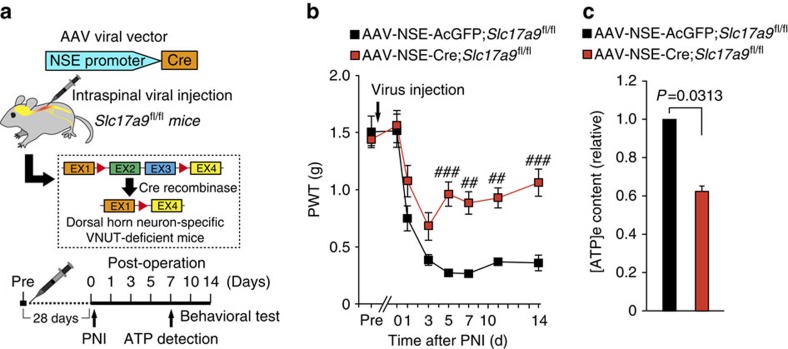
VNUT expressed in SDH neurons is required for increased spinal extracellular ATP and pain hypersensitivity after PNI. (**a**) Schematic diagram of the experimental protocol. (**b**) PWT of *Slc17a9*^fl/fl^ mice intraspinally injected with AAV-NSE-AcGFP or AAV-NSE-Cre viral vectors before and after PNI (AAV-NSE-AcGFP: *n=*5, AAV-NSE-Cre: *n=*8; ^##^*P<*0.01, ^###^*P<*0.001 versus AAV-NSE-AcGFP;*Slc17a9*^fl/fl^, two-way ANOVA with *post hoc* Bonferroni test). (**c**) Measurement of [ATP]_e_ content in the ACSF media of spinal cord slices isolated from *Slc17a9*^fl/fl^ mice intraspinally injected with AAV-NSE-AcGFP or AAV-NSE-Cre viral vectors 7 days after PNI (*n=*6, Wilcoxon matched-pairs signed rank test). Values are means±s.e.m.

We next investigated the possible involvement of VNUT in SDH neurons in exocytotic ATP release and neuropathic pain. We injected an AAV vector encoding Cre under the control of a human neuron-specific enolase (NSE) promoter (AAV-NSE-Cre) into the unilateral SDH of R26-tdTomato mice. Strong tdTomato fluorescent signals were observed on the injected side of the SDH (Supplementary Fig. 5a). These signals were restricted to cells labelled with neuronal nuclei (NeuN), but not with Iba1, GFAP or adenomatous polyposis coli protein (APC; oligodendrocytic marker) (Supplementary Fig. 5b). Strikingly, there were no tdTomato signals in DRG neurons (0 of 1,348 Nissl^+^ neurons, Supplementary Fig. 5c). These results indicate that Cre activity in the mice injected with AAV-NSE-Cre into the SDH is highly specific to SDH neurons. Then, to knock out VNUT in SDH neurons, we injected the AAV-NSE-Cre into the SDH of *Slc17a9*^fl/fl^ mice (Fig. 6a). Behavioural analysis showed that there were no behavioural differences in normal sensory perception between AAV-NSE-Cre;*Slc17a9*^fl/fl^ mice (1.55±0.14 g, *n*=8 mice) and their AAV-NSE-AcGFP;*Slc17a9*^fl/fl^ controls (1.52±0.15 g, *n*=5 mice; *P*>0.05). Interestingly, following PNI, AAV-NSE-AcGFP;*Slc17a9*^fl/fl^ mice showed long-lasting tactile allodynia (Fig. 6b), whereas AAV-NSE-Cre;*Slc17a9*^fl/fl^ mice exhibited a reversal of pain behaviour (Fig. 6b). Consistently, [ATP]_e_ levels within the spinal cord were significantly lower in AAV-NSE-Cre;*Slc17a9*^fl/fl^ mice compared with AAV-NSE-AcGFP;*Slc17a9*^fl/fl^ controls (*P=*0.0313, Fig. 6c). In addition, we found similar attenuation phenotypes of spinal [ATP]_e_ content and PNI-induced allodynia in *Slc17a9*^fl/fl^ mice with Cre expression in SDH neurons by using AAV-ESYN-Cre (Supplementary Figs 6 and 7). These results indicate that VNUT in SDH neurons is crucial for enhanced exocytotic ATP release within the spinal cord and tactile allodynia after PNI.

### Phenotypic rescue by VNUT transduction of SDH neurons

**Figure 7:**
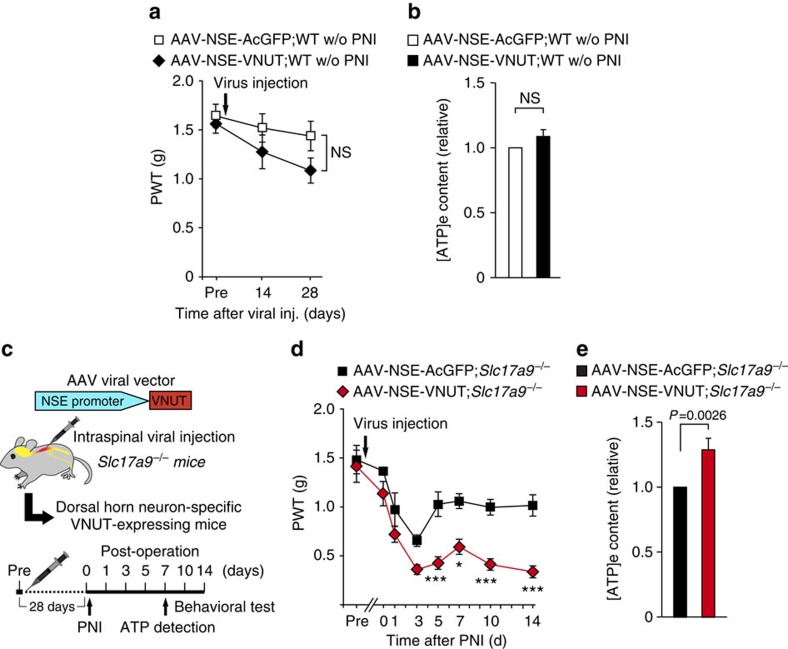
Ectopic expression of VNUT in SDH neurons of *Slc17a9*^−/−^ mice rescues tactile allodynia and enhances spinal ATP after PNI. (**a**) PWT of WT mice before (pre) and after intraspinal injection of AAV-NSE-AcGFP or AAV-NSE-VNUT viral vectors without (w/o) PNI (*n=6*, two-way ANOVA with *post hoc* Bonferroni test). (**b**) Measurement of [ATP]_e_ content in the ACSF media of spinal cord slices isolated from WT mice 28 days after intraspinal injection with AAV-NSE-AcGFP or AAV-NSE-VNUT viral vectors without PNI (*n=13*, Wilcoxon matched-pairs signed rank test). (**c**) Schematic diagram of experimental protocol for **d**,**e**. (**d**) PWT of *Slc17a9*^*−/−*^ mice intraspinally injected with AAV-NSE-AcGFP or AAV-NSE-VNUT viral vectors before and after PNI (AAV-NSE-AcGFP; *Slc17a9*^*−/−*^: *n=5*, AAV-NSE-VNUT; *Slc17a9*^*−/−*^: *n=6*; *P<0.05, ***P<0.001 versus AAV-NSE-AcGFP;*Slc17a9*^*−/−*^, two-way ANOVA with *post hoc* Bonferroni test). (**e**) Measurement of [ATP]_e_ content in the ACSF media of spinal cord slices isolated from *Slc17a9*^*−/−*^ mice intraspinally injected with AAV-NSE-AcGFP or AAV-NSE-VNUT viral vectors 7 days after PNI (*n=*14, Wilcoxon matched-pairs signed rank test). Values are means±s.e.m. NS, not significant.

To confirm the importance of VNUT expression in SDH neurons for tactile allodynia, we undertook AAV-mediated VNUT gene transduction. We cloned the mouse *Slc17a9* gene from the spinal cord of WT mice, and placed it into an AAV2/9 vector under the control of the NSE promoter (AAV-NSE-VNUT). This construct worked well *in vitr*o, as ATP release was increased in the Neuro2A neuronal cell line transduced with this vector (Supplementary Fig. 8a,b). Intraspinal AAV-NSE-VNUT injection successfully induced *Slc17a9* genes in the spinal cord of naive WT mice with no effect on the DRG (Supplementary Fig. 8c). Contrary to our expectation, these mice did not show pain hypersensitivity (Fig. 7a) and only a slight increase in spinal [ATP]_e_ content (Fig. 7b). These results indicate that the upregulation of VNUT in SDH neurons of normal mice is not sufficient to produce tactile allodynia.

Next, we intraspinally injected the AAV-NSE-VNUT into *Slc17a9*^*−/−*^ mice (Fig. 7c). Importantly, ectopic expression of *Slc17a9* in SDH neurons of *Slc17a9*^*−/−*^ mice successfully rescued the defects in PNI-induced tactile allodynia (Fig. 7d) and enhancement of spinal [ATP]_e_ levels (Fig. 7e). Another construct (AAV-ESYN-VNUT) conferred a similar effect on pain behaviour (Supplementary Fig. 8d). Taken together, these results strongly support our concept that VNUT expressed in SDH neurons plays a central role in enhanced ATP release within the spinal cord and full manifestation of neuropathic pain after PNI.

## Discussion

A large body of work has accumulated over the last decade indicating that purinergic receptors for extracellular ATP are essential for the pain hypersensitivity^15^,^16^,^17^,^18^,^20^. This work has led to two key unresolved questions: one is the release mechanism for the ATP that causes pain hypersensitivity and the other is the cell type that releases this pain-producing ATP^9^,^22^. In the present study, we demonstrate that VNUT in SDH neurons contributes to the PNI-induced enhancement of exocytotic ATP release within the spinal cord and is a widespread player for producing tactile allodynia after PNI. This implies that SDH neurons are the principal source of extracellular ATP in the spinal cord that causes PNI-induced pain hypersensitivity. To the best of our knowledge, our results show for the first time the causal role of VNUT in neuropathic pain, and therefore may provide a novel therapeutic target.

Increased expression of microglial ATP receptors including P2X4R and P2Y_12_R is crucial for the pathogenesis of neuropathic pain^9^,^15^,^17^. In addition, ATP level in the cerebrospinal fluid is not changed after PNI^15^, hence it has long been thought that the constitutive level of [ATP]_e_ is sufficient to cause ongoing activation of these ATP receptors, leading to tactile allodynia. However, our detailed analysis shows that [ATP]_e_ level within the spinal cord is concomitantly increased after PNI, and is dependent on VNUT. Furthermore, *Slc17a9*^*−/−*^ mice exhibited less allodynic behaviour compared with WT mice, despite the fact that the expression levels of P2X4R and P2Y_12_R in the spinal cord were not affected by VNUT deficiency. Therefore, our results may provide the novel concept that enhancement of both ATP receptor expression and extracellular ATP release within the spinal cord is necessary for full expression of neuropathic pain.

An unexpected finding in the present study was, however, the partial recovery from pain hypersensitivity in *Slc17a9*^*−/−*^ mice and after siRNA-mediated knockdown of spinal VNUT expression. Particularly at an early-time point after PNI (that is, 3 days post-PNI) where expression of VNUT in the spinal cord is not significantly increased, deletion of VNUT expression produced only a minimal effect on reversal of pain hypersensitivity, suggesting the involvement of a VNUT-independent mechanism (for example, hemichannels^41^,^42^) on the onset of neuropathic pain. In addition, the fact that the PNI-induced enhancement of spinal [ATP]_e_ content on day 7 was almost completely suppressed in *Slc17a9*^*−/−*^ mice might suggest that even constitutive level of [ATP]_e_ may has potential for activating microglial ATP receptors, to a lesser extent, to produce an abnormality in pain sensation. Alternatively, ATP-independent mechanisms may also be involved^8^,^43^,^44^.

It was surprising that the PNI-induced increase in spinal [ATP]_e_ concentration was not observed on 28 days. In light of the expression pattern of VNUT in the spinal cord after PNI, it seems plausible that the enhancement of spinal [ATP]_e_ content is dependent on the upregulation of VNUT expression. However, the strong attenuation of tactile allodynia in *Slc17a9*^*−/−*^ mice lasted for more than 1 month following PNI. Our results suggest that VNUT is necessary for both early and late phases of pain hypersensitivity after PNI, but increased spinal [ATP]_e_ content is only present in early but not late phase. However, these results may appear to produce an apparent contradiction. One possibility is that the lack of VNUT expression during the early phase may influence PNI-induced long-term alterations crucial for pain chronicity, for example, the PNI-induced sensitization or alterations of pain pathway along the pain neuraxis, from peripheral nociceptors and spinal cord to supraspinal brain regions^21^,^44^,^45^. However, it remains unknown and will need further investigations.

Following PNI, *Slc17a9*^*−/−*^ mice exhibited distinctive phenotypes, including attenuated symptom of tactile allodynia and lower level of [ATP]_e_ within the spinal cord compared with WT mice. We therefore regarded these phenotypes as read-outs of deletion of specific VNUT responsible for extracellular ATP release and neuropathic pain. Notably, among a variety of conditional knockout mice used in this study, only the mice lacking VNUT in SDH neurons phenocopied those of *Slc17a9*^*−/−*^ mice. Combined with results from the rescue experiments whereby the transduction of SDH neurons in *Slc17a9*^*−/−*^ mice with VNUT caused tactile allodynia and increased [ATP]_e_ content within the spinal cord, we suggest that VNUT-expressing SDH neurons are a source of extracellular ATP within the spinal cord that causes pain hypersensitivity after PNI. Although it has previously been reported that a subset of cultured GABAergic spinal neurons release ATP simultaneously with GABA^46^, the subpopulation of SDH neurons that expresses VNUT and is responsible for ATP release and neuropathic pain remains to be elucidated. This would be an important question to be addressed in future studies.

In our experiments with WT mice injected with AAV-NSE-VNUT, ectopic expression of VNUT in SDH neurons of normal WT mice *per se* unexpectedly gave rise to a faint increase in spinal [ATP]_e_ content. In addition, VNUT transduction of SDH neurons did not cause pain hypersensitivity in the absence of PNI. These results may imply that PNI-induced machineries, such as additive stimuli that elicit ATP exocytosis from neurons (for example, signals that evoke Ca^2+^ release from ER store in SDH neurons), and/or microglial activation accompanied by enhanced expression of P2X4R^15^ or P2Y_12_R^17^, may be required.

Our previous studies have shown that reactive microglia and astrocytes in the SDH play crucial roles in producing tactile allodynia after PNI^12^,^47^, which raises the possibility that signals from reactive glial cells might participate in VNUT upregulation in the spinal cord after PNI. However, neither mice lacking interferon regulatory factor-8 (IRF8), a crucial factor for microglial activation^12^, nor mice with astrocyte-specific deficiency of signal transducer and activator of transcription 3 (STAT3), crucial for astrocytic activation^48^, showed any attenuation in PNI-induced VNUT expression in the spinal cord (data not shown), suggesting the possible involvement of other mechanisms (for example, signals from injured primary afferent). Although elucidating this issue needs further investigations, suppression of upregulated spinal VNUT expression by siRNAs reversed tactile hypersensitivity. Furthermore, *Slc17a9*^*−/−*^ mice showed attenuated symptoms of neuropathic pain after PNI without affecting normal sensory processing. Therefore, interfering the expression or function of VNUT may be a novel therapeutic strategy for neuropathic pain, for which there is currently no effective treatment.

As for the mechanism of ATP release from the broad range of cell types, two mechanistically distinct pathways have mainly been defined^6^: first is exocytosis of ATP-containing vesicles, and second is efflux of cytosolic ATP via plasma membrane channels, such as connexin or pannexin hemichannels. Our data suggest that exocytosis of VNUT-expressing vesicles from SDH neurons is critical for the PNI-induced increase in [ATP]_e_ content within the spinal cord and pain hypersensitivity. Recent studies have also shown crucial roles of both connexin-43 (ref. 41) and pannexin-1 (ref. 42) hemichannels in the spinal cord or DRG neurons for neuropathic pain. Although it is yet to be revealed whether these hemichannels regulate [ATP]_e_ level in the spinal cord, VNUT and these hemichannels may be concertedly involved in the enhancement of spinal [ATP]_e_ level via an unknown mechanism, thereby producing neuropathic pain. Further investigations are warranted to fully understand the mechanisms of extracellular ATP supply within the spinal cord. In addition, ATP has also been implicated in inflammatory pain caused by intraplantar CFA injection through the activation of P2X3 receptors in DRG neurons^49^,^50^. However, our results show that VNUT deficiency did not affect CFA-induced pain behaviour. Thus, a VNUT-independent mechanism (for example, hemichannels) may be involved in the pathogenesis of inflammatory pain. In this study, [ATP]_e_ was detected even in *Slc17a9*^*−/−*^ spinal cord after PNI. Given that the equivalent level of [ATP]_e_ was observed in naive WT spinal cord, it is assumed that preparation of tissue slices itself can lead to a leak or release of ATP, to some extent, from the cells that reside on the surface of slices, although a possible involvement of other mechanisms than exocytotic ATP release cannot be excluded.

In conclusion, we show that VNUT-dependent exocytic ATP release from SDH neurons is a crucial mechanism for neuropathic pain. Thus, our present study not only provides conceptual advances regarding the pathogenesis of neuropathic pain, but an insight into the role of VNUT-dependent exocytotic ATP release in CNS disorders. Extracellular ATP released through as-yet-unknown mechanism is crucial for diverse diseases^7^,^38^, therefore our findings may provide impetus to better understand the contribution of ATP in disease pathologies.

## Methods

### Animals

Male VNUT-deficient (*Slc17a9*^*−/−*^) mice^25^,^26^, and their wild-type littermates, and C57BL/6 mice (Clea, Japan) were used. Male and female *Slc17a9*-floxed mice (B6.129-*Slc17a9*^*tm1.1Rpa*^/J), *Cx3cr1-Cre*^*ERT2*^ transgenic mice (B6.129P2(C)-*Cx3cr1*^*tm2.1(cre/ERT2)Jung*^/J), *Gfap-Cre* transgenic mice (B6.Cg-Tg(*Gfap-cre*)73.12Mvs/J) and Rosa26-tdTomato reporter mice (B6.Cg-Gt(ROSA)26Sortm14(CAG-tdTomato)Hze/J) were purchased from Jackson Laboratory (USA). *Cd11b-Cre* transgenic mice (B6.Cg-Tg(*Itgam-cre*)^2781Gkl/Flmg^)^51^ were kindly provided by Prof. George Kollias (Biomedical Sciences Research Centre ‘Alexander Fleming'). For induction of Cre recombinase, 4–6-week-old *Cx3cr1-Cre*^*ERT2*^ mice were injected s.c. with 2 mg tamoxifen (Sigma, USA) solved in 100 μl corn oil (Wako, Japan) once a day for 2 days. All mice used were aged 8–15 weeks at the start of each experiment, and were housed individually and in groups of two or three per cage at a temperature of 22±1 °C with a 12-h light–dark cycle, and were fed food and water ad libitum. Genotyping was performed as previously described. All experimental procedures were performed under the guidelines of Kyushu University.

### Peripheral nerve injury

We used the spinal nerve injury model^52^ with some modifications^12^. Briefly, under isoflurane (2%) anaesthesia, a small incision at L3–S1 was made. The paraspinal muscle and fat were removed from the L5 traverse process, which exposed the parallel-lying L3 and L4 spinal nerves. The L4 nerve was then carefully isolated and cut. The wound and the surrounding skin were sutured with 5-0 silk.

### Measurement of extracellular ATP

A previously described procedure for tissue ATP measurement^7^ was used with some modifications. Mice were deeply anaesthetized with pentobarbital. Following spinal laminectomy, the L3–L4 spinal cord was removed immediately. The tissues were vertically separated at the median, and hemisections of the spinal cord were subjected to extracellular ATP measurement. Acute spinal cord slices were prepared using a VT1200 vibrating blade microtome (Leica, Germany), and incubated in ice-cold ACSF (125 mM NaCl, 3.5 mM KCl, 2.0 mM CaCl_2_, 2.0 mM MgSO_4_, 1.25 mM KH_2_PO_4_, 26 mM NaHCO_3_, 10 mM D-glucose) with the ectonuclease inhibitor ARL67156 (100 μM) (Sigma) for 18 min, with/without tetanus toxin (2 μM; Sigma) or thapsigargin (10 μM; Wako). Then the ACSF was collected and ATP levels were determined using an ATP determination kit (Molecular Probes, USA). Luminescence was measured by a Mithras LB940 multi-label microplate reader (Berthold Technologies, Germany). For normalization, protein amounts of each sample were measured by bicinchoninic acid assay (Thermo Fisher Scientific, USA).

### Quantitative real-time PCR

Mice were deeply anaesthetized with pentobarbital, perfused transcardially with phosphate-buffered saline (PBS) and the L3–L4 spinal cord was immediately removed. The tissues were vertically separated and hemisections of the SDH were subjected to total RNA extraction using Trisure (Bioline, USA), according to the manufacturer's protocol, and purified with the RNeasy mini plus kit (Qiagen, USA). The amount of total RNA was quantified by measuring OD_260_ using a Nanodrop spectrophotometer (Nanodrop, Wilmington, DE). For reverse transcription, 150 ng of total RNA was transferred to the reaction with Prime Script reverse transcriptase (Takara, Japan). Quantitative PCR was performed with FastStart Essential DNA Probes Master (Roche, Switzerland) using a LightCycler 96 (Roche, Switzerland). Expression levels were normalized to the values for 18S ribosomal RNA. The sequences of TaqMan primer pairs and probe are described below.

P2X4R: 5′-ACAACGTGTCTCCTGGCTACAAT-3′ (forward), 5′-GTCAAACTTGCCAGCCTTTCC-3′ (reverse), 5′-FAM-CAATGAGCAACGCACACTCACCAAGG-TAMRA-3′ (probe)

P2Y12R: 5′-TGAAGACCACCAGGCCATTT-3′ (forward), 5′-AGGCCCAGATGACAACAGAAA-3′ (reverse), 5′-FAM-AAACGTCCAGCCCCAGCAATCTCTTG-TAMRA-3′ (probe)

VNUT (*Slc17a9*): 5′-TGCTGGCAATTCCTGCTAGTCT-3′ (forward), 5′-GTGATGACTCTGTAACCCTGACTGA-3′ (reverse), 5′-FAM-TCAGTGGGTTCATCTCCGACCGC-TAMRA-3′ (probe)

Iba1 (*Aif1*): 5′-GATTTGCAGGGAGGAAAAGCT-3′ (forward), 5′-AACCCCAAGTTTCTCCAGCAT-3′ (reverse), 5′-FAM-CAGGAAGAGAGGCTGGAGGGGATCAA-TAMRA-3′ (probe)

The primers and probe for 18S were obtained from Applied Biosystems (USA).

### Immunohistochemistry

Mice were deeply anesthetized by pentobarbital and perfused transcardially with PBS followed by ice-cold 4% paraformaldehyde/PBS. Transverse L4 spinal cord sections (30 μm) and L4 DRG sections (15 μm) were incubated for 48 h at 4 °C with primary antibody for CD11b (1:1,000, Serotec, USA), Iba1 (1:2,000, Wako), GFAP (1:500, Chemicon, USA), NeuN (1:200, Chemicon), APC (1:200, Calbiochem, USA), NF200 (neurofilament 200, 1:400), P2X3R (1:2,500, Chemicon), IB4 (isolectin B4)-Alexa488 (1:200, Invitrogen, USA) and PKC-γ (protein kinase C gamma, 1:5,000, Santa Cruz, USA). Tissue sections were incubated with secondary antibodies conjugated to Alexa Fluor 405, 488 or 546 (1:1,000, Molecular Probes) and mounted with VECTASHIELD with or without DAPI (Vector Laboratories, USA). Three to five sections from the L4 spinal cord segments or L4 DRG segments of each mouse were randomly selected and analysed using an LSM510 Imaging System (Carl Zeiss, Japan).

### Vector construction and AAV virus production

Each AAV expression plasmid pZac2.1 (Penn Vector, USA) carrying AcGFP, venus, Cre-Ires-AcGFP cassette, Cre-Ires-Venus cassette or VNUT-Ires-AcGFP cassette driven by the neuron-specific enolase (NSE) or the ESYN promoters was constructed to target neurons. A fragment that includes mouse VNUT (*Slc17a9)* was amplified by PCR with primers (forward: 5′-CATGTCTAGATGCCATCCCAGCGCT-3′, reverse: 5′-CATGGGATCCTTAGAGGTCCTCATGAGTGGGG-3′) using a cDNA collection of WT mice spinal cord, and was inserted into pZac2.1. Viral vectors were generated by triple transfection of HEK293T cells with each AAV expression plasmid pZac2.1, the pAAV2/9 plasmid (Penn Vector) and the adenovirus helper plasmid pAdΔF6 (Penn Vector) using polyethyleneimine. Viral lysate was harvested at 72 h post-transfection and lysed by freeze-and-thaw cycles. The viral lysate was purified through two rounds of CsCl ultracentrifugation, and then concentrated using Amicon ultra centrifugal filter units (Millipore, Germany). Genomic titre was determined by measurement of vector genome copies using Pico Green fluorometric reagent (Molecular Probes). Vectors were stored in aliquots at −80 °C until use.

### AAV viral injections

Mice aged 7–8 weeks were anaesthetized with ketamine and xylazine (100 mg and 10 mg kg^−1^, respectively), and mounted into a custom-made stereotaxic frame. Paraspinal muscles around the left side of the interspace between Th13 and L1 vertebrae were removed, and the dura mater and the arachnoid membrane were carefully incised using the tip of a 30 G needle to make a small window to allow the microcapillary Femtotip II(Eppendorf, Germany) insert directly into the SDH. The microcapillary filled with each viral vector (1 × 10^12^ GC per ml for Fig. 7, Supplementary Fig. 7, or 5 × 10^12^ GC per ml for Fig. 6, Supplementary Figs 4–6) was inserted into the SDH (250 μm in depth from the surface of the dorsal root entry zone) through the small window, and was pressure-ejected (600 hPa) for 30 s (∼0.2–0.3 μl injection) using the FemtoJet Express microinjector (Eppendorf). After microinjection, the inserted microcapillary was removed, followed by suturing the skin with 5-0 silk. Following surgery, animals were returned to their home cages for at least 4 weeks before being used for analysis. For targeting DRG neurons, neonate mice (postnatal day 0 or 1) were injected i.p. with a volume of 20 μl (1.0 × 10^13^ GC per ml) of each viral vector, as described in a previous report^40^, with some modification.

### Intrathecal catheterization

Under isoflurane (2%) anaesthesia, mice were implanted with a 32-gauge intrathecal catheter (ReCathCo, Allison Park, PA) through the atlanto-occipital region and in the lumbar enlargement (close to L3–L4 segments) of the spinal cord^12^,^53^. After 7 days of recovery, the catheter placement was verified by the observation of transient hindpaw paralysis induced by intrathecal injection of lidocaine (2%, 1.5 μl). Animals that failed to show any paralysis were not used in experiments. Intrathecal injection of VNUT stealth siRNA or scrambled control RNA (10 pmol per 2.5 μl) was followed by infusion of 3 μl of PBS. Sequences of the VNUT stealth (Invitrogen) siRNAs and the non-targeting scramble siRNA used in this study were as follows.

VNUT siRNA-1 sense sequence: 5′-GAUGCUGGCAAUUCCUGCUAGUCUA-3′.

VNUT siRNA-1 antisense sequence: 5′-UAGACUAGCAGGAAUUGCCAGCAUC-3′.

VNUT siRNA-2 sense sequence: 5′-CAAGGCUAUGAUCUUUGCAUCAGCU-3′.

VNUT siRNA-2 antisense sequence: 5′-AGCUGAUGCAAAGAUCAUAGCCUUG-3′.

Scramble siRNA sense sequence: 5′-CAGUGGAGGCGUCUUUACUCGAUCA-3′.

Scramble siRNA antisense sequence: 5′-UGAUCGAGUAAAGACGCCUCCACUG-3′.

### Behavioural studies

To assess mechanical sensitivity, calibrated von Frey filaments (0.02–2.0 g, North Coast Medical, USA) were applied to the plantar surfaces of the hindpaws of mice with or without PNI, or peripheral inflammation caused by intraplantar injection of CFA (0.01 mg per 20 μl) and the 50% PWT was determined. The volume of hindpaw oedema was measured by weighing the hindpaw. For the hot-plate test, mice were placed on a metal surface maintained at 45, 49 or 52 °C within a 25-cm-high Plexiglass box (25 × 2 cm). The latency to either lick the hindpaw or jump was recorded as a nocifensive end point^54^. Noxious heat-evoked tail-flick responses were measured following the application of radiant heat (Ugo Basile, Italy) to the tail. The intensity of the heat stimulus was adjusted to 50 V, and the latency of the tail withdrawal response (seconds) was measured^16^. In an acetone test, acetone (20 μl) was applied through wire mesh flooring onto the plantar surface of the left hindpaw to produce evaporative cooling, and behaviour was observed during the first 30 s after acetone application and was scored by adding together each of five acetone applications (0, no response; 1, licking or flinching; 2, strong flinching or licking).

### Statistics

Statistical significance was determined using the Wilcoxon matched-pairs signed rank test (Figs 6c and 7b,e), two-way ANOVA with *post hoc* Bonferroni test (Figs 2a,g, 3a,d–f and 5, Figs 6b and 7a,d) or one-way ANOVA with *post hoc* Tukey Multiple Comparison test (Figs 1b–d and 2f,h, Figs 3c and 4b,c) using GraphPad Prism 5.04 software. Differences were considered significant at *P<*0.05.

### Data availability

The data that support the findings of this study are available from the corresponding author upon reasonable request.

## Additional information

**How to cite this article:** Masuda, T. *et al.* Dorsal horn neurons release extracellular ATP in a VNUT-dependent manner that underlies neuropathic pain. *Nat. Commun.* 7:12529 doi: 10.1038/ncomms12529 (2016).

## Supplementary Material

Supplementary Information Supplementary Figures 1-8
