# Supplementary material for: Dorsal horn neurons release extracellular ATP in a VNUT-dependent manner that underlies neuropathic pain
Source: Nat Commun. 2016 Aug 12;7:12529. doi: 10.1038/ncomms12529 (PMC4990655; doi:10.1038/ncomms12529)
Supplement: Supplementary Information — Supplementary Figures 1-8 [file ncomms12529-s1.pdf]

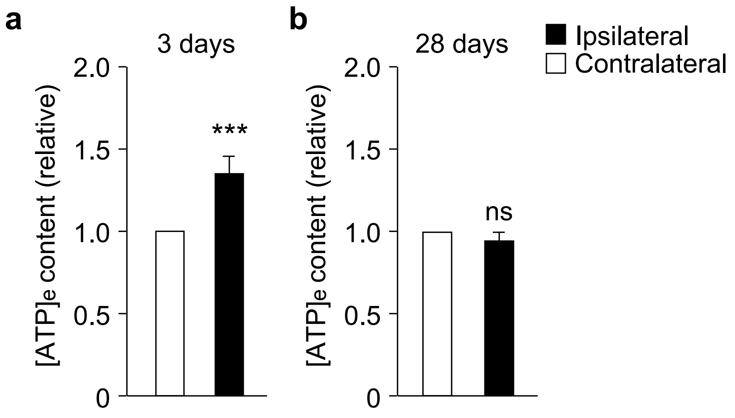

Supplementary Figure 1. PNI increases an extracellular ATP level within the spinal cord 3 days, but not 28 days, after PNI.
Measurement of $[ATP]_e$ content in the ACSF media of ipsilateral and contralateral spinal cord slices taken from wild-type mice (**a**) 3 days and (**b**) 28 days after PNI. Values represent the relative ratio of ATP levels to the contralateral side (**a**, *n=13;* **b***, n=8*; ****P=0.0007* vs. Contralateral, Wilcoxon matched-pairs signed rank test). Values are means ± s.e.m.

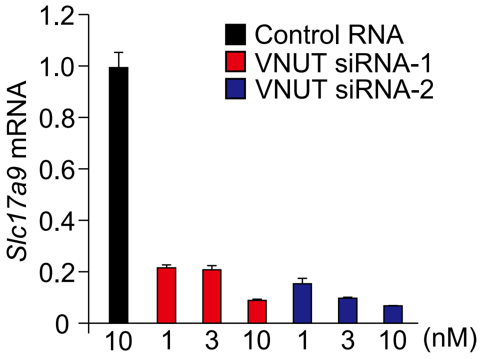

Supplementary Figure 2. Effect of siRNAs on VNUT expression.
Real-time PCR analysis of *Slc17a9* mRNA in BV2 cells treated with control RNA (10 nM) or VNUT siRNAs (1, 3, 10 nM) for 48 h. Values represent the relative ratio of *Slc17a9* mRNA (normalized to the value for *18s* mRNA) to the control RNA (*n=3*). Values are means ± s.e.m.

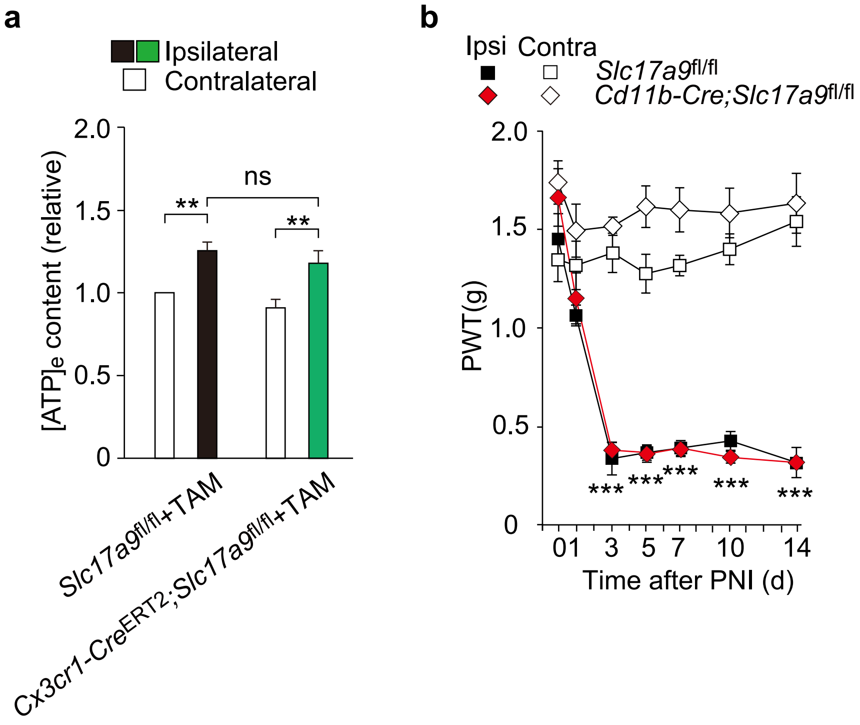

Supplementary Figure 3. Microglial VNUT is not required for PNI-induced increase in extracellular ATP within the spinal cord and pain hypersensitivity.
(a) Measurement of $[ATP]_e$ content in the ACSF media of spinal cord slices isolated from *Slc17a9*$^{fl/fl}$ or *Cx3cr1-Cre*^{ERT2};*Slc17a9*$^{fl/fl}$ mice that had been injected with tamoxifen (TAM), 7 days after PNI (*n=14*; ***P<0.01*, one-way ANOVA with post hoc Tukey Multiple Comparison test). **(b)** PWT of microglia-specific VNUT conditional knockout (*Cd11b-Cre;Slc17a9*$^{fl/fl}$) mice and their *Slc17a9*$^{fl/fl}$ littermate controls before and after PNI [*Slc17a9*$^{fl/fl}$: *n=5*, *Cd11b-Cre;Slc17a9*$^{fl/fl}$: *n=6*; ****P<0.001* vs. Contra (*Slc17a9*$^{fl/fl}$), two-way ANOVA with post hoc Bonferroni test]. Values are means ± s.e.m.

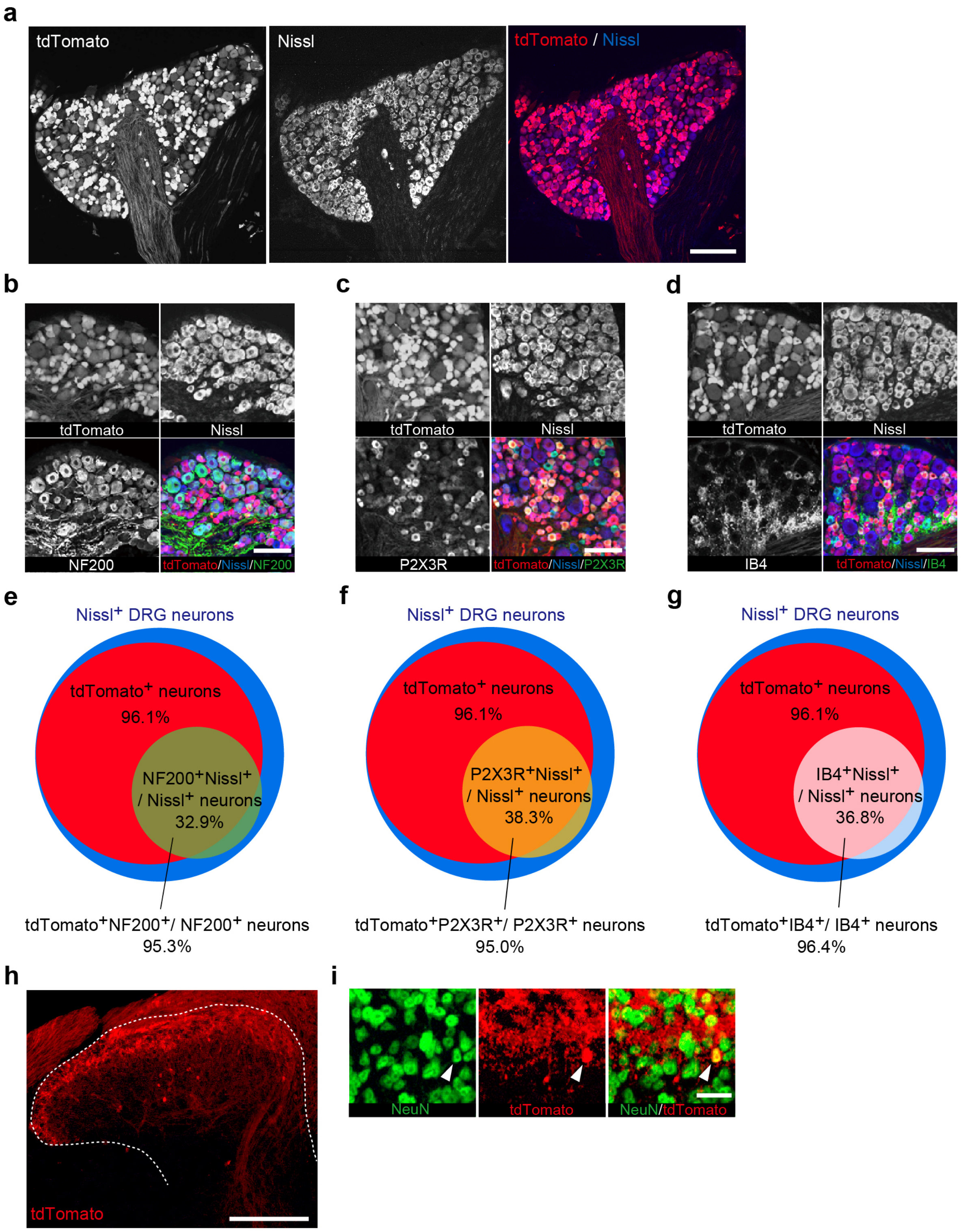

Supplementary Figure 4. AAV-mediated Cre recombination in DRG neurons.
(a) Representative images (of three experiments) showing tdTomato fluorescence and Nissl staining in the L4 DRG of ROSA26 (R26)-tdTomato reporter mice intraperitoneally injected with AAV-ESYN-Cre vector. (scale bar, 200 μm) **(b-d)** Representative images (of three experiments) showing fluorescent signals of tdTomato with Nissl staining and immunofluorescence labelling of **(b)** NF200, **(c)** P2X3R or **(d)** IB4 in the L4 DRG of R26-tdTomato mice treated with AAV-ESYN-Cre vector. (scale bar, 100 μm) **(e-g)** Charts summerize the results of Supplementary Figures 3b-d showing the distribution of tdTomato-positive DRG neurons. **(h)** Representative images (of three experiments) showing tdTomato fluorescence in the L4 spinal cord of R26-tdTomato mice intraperitoneally injected with AAV-ESYN-Cre vector. (scale bar, 200 μm) **(i)** Fluorescent signals of tdTomato and immunofluorescence labelling of NeuN in the L4 spinal cord of R26-tdTomato mice treated with AAV-ESYN-Cre vector. Arrowhead indicates colocalization of tdTomato signal with NeuN signal. (scale bar, 20 μm)

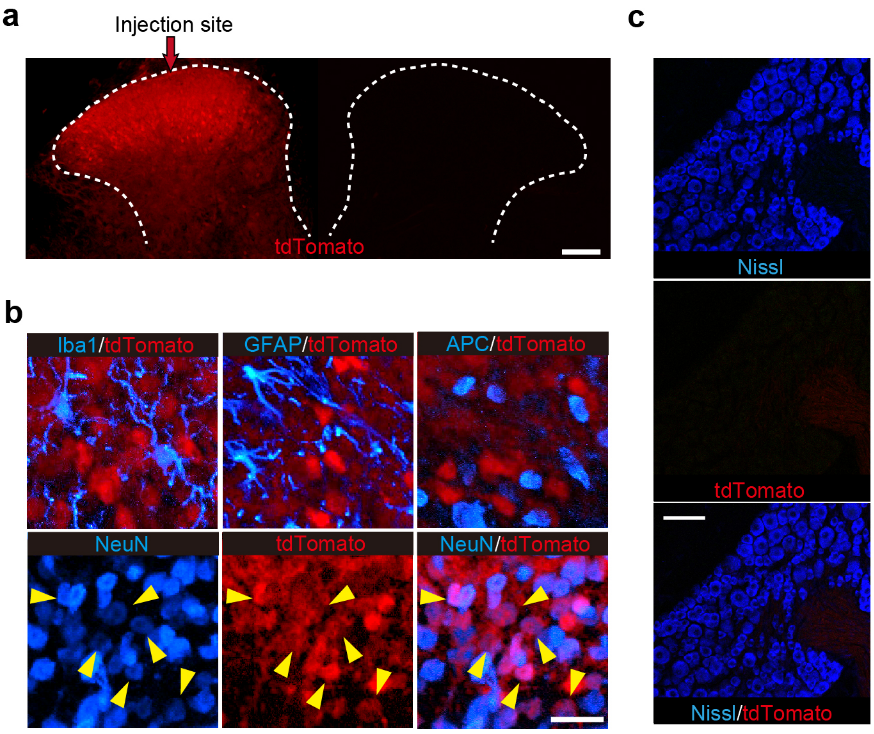

Supplementary Figure 5. AAV-mediated Cre recombination in spinal dorsal horn neurons.

(a) Representative images (of three experiments) showing tdTomato fluorescence in the L4 spinal cord of R26-tdTomato mice intraspinally injected with AAV-NSE-Cre vector. (scale bar, 100 μm) **(b)** Representative images (of three experiments) showing tdTomato fluorescence with immunofluorescent labelling of Iba1 (microglia), GFAP (astrocyte), APC (oligodendrocyte) or NeuN (neuron) in the L4 spinal cord of R26-tdTomato mice treated with AAV-NSE-Cre vector. Arrowheads indicate colocalization of tdTomato signals with NeuN signals. (scale bar, 20 μm) **(c)** Representative images (of three experiments) showing Nissl staining with tdTomato fluorescence in the L4 DRG of R26-tdTomato mice treated with AAV-NSE-Cre vector. (scale bar, 100 μm)

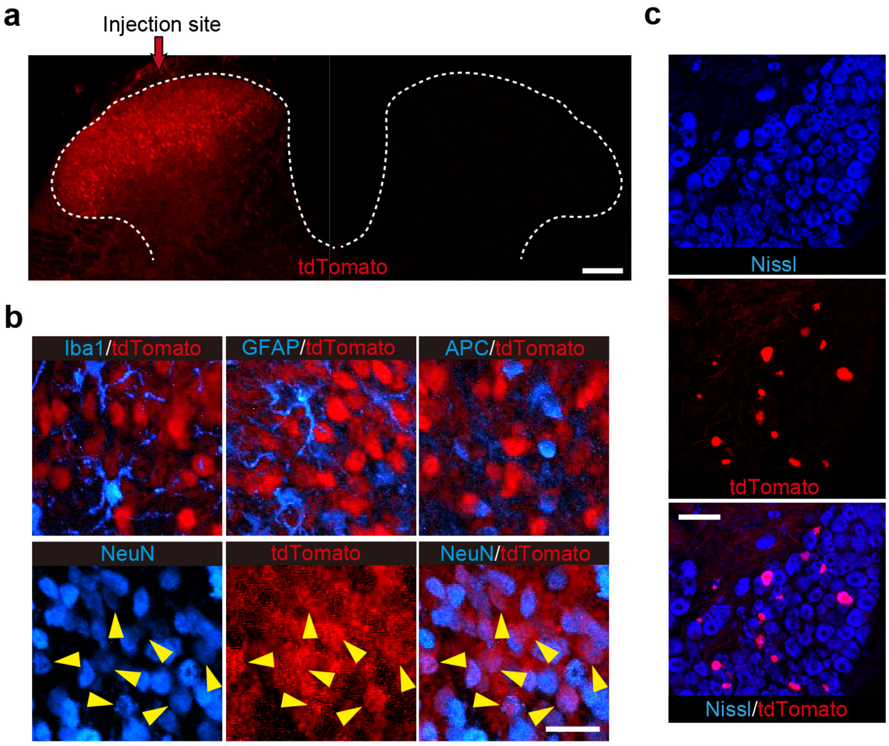

Supplementary Figure 6. AAV-mediated Cre recombination in spinal dorsal horn neurons.

(a) Representative images (of three experiments) showing tdTomato fluorescence in the L4 spinal cord of R26-tdTomato mice intraspinally injected with AAV-ESYN-Cre vector. (scale bar, 100 μm) **(b)** Representative images (of three experiments) showing tdTomato fluorescence with immunofluorescent labelling of Iba1 (microglia), GFAP (astrocyte), APC (oligodendrocyte) or NeuN (neuron) in the L4 spinal cord of R26-tdTomato reporter mice treated with AAV-ESYN-Cre vector. Arrowheads indicate colocalization of tdTomato signals with NeuN signals. (scale bar, 20 μm) **(c)** Representative images (of three experiments) showing Nissl staining with tdTomato fluorescence in the L4 DRG of R26-tdTomato mice treated with AAV-ESYN-Cre vector. A small number of Nissl-positive DRG neurons was positive to tdTomato (8.47%: 103 of 1216 Nissl^{+} cells). (scale bar, 100 μm)

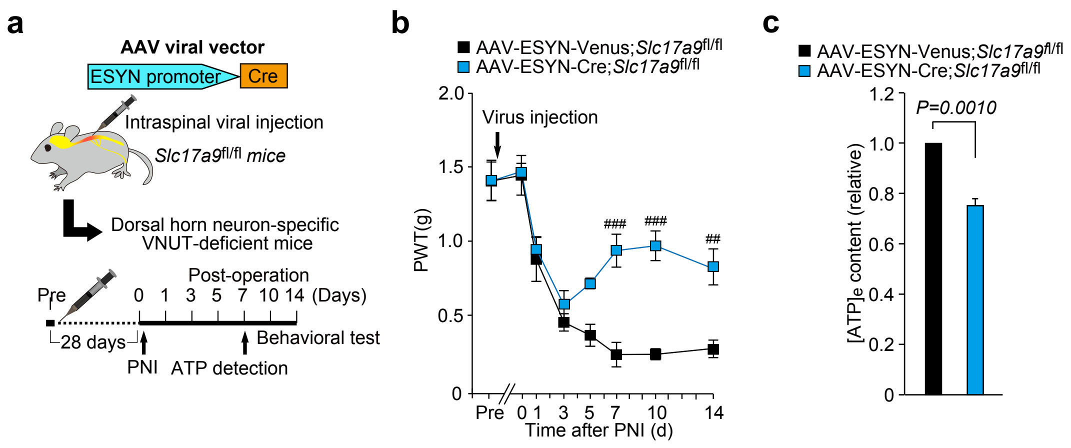

Supplementary Figure 7. VNUT expressed in SDH neurons is involved in increased spinal ATP supply and pain hypersensitivity after PNI.

(a) Schematic diagram of the experimental protocol. **(b)** PWT of *Slc17a9*$^{fl/fl}$ mice intraspinally injected with AAV-ESYN-Venus or an AAV-ESYN-Cre viral vectors before and after PNI (AAV-ESYN-Venus;*Slc17a9*$^{fl/fl}$: *n*=5, AAV-ESYN-Cre;*Slc17a9*$^{fl/fl}$: *n=6*; ##*P<0.01*, ###*P<0.001* vs. AAV-ESYN-Venus;*Slc17a9*$^{fl/fl}$, two-way ANOVA with post hoc Bonferroni test) **(c)** Measurement of $[ATP]_e$ content in the ACSF media of spinal cord slices isolated from *Slc17a9*$^{fl/fl}$ mice intraspinally injected with AAV-ESYN-Venus or AAV-ESYN-Cre vectors 7 days after PNI (*n=11*, Wilcoxon matched-pairs signed rank test).

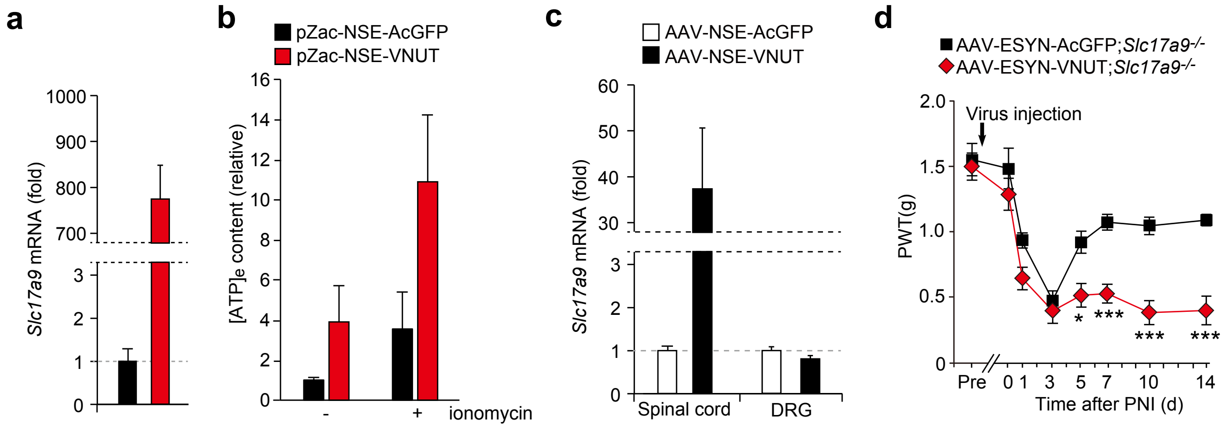

Supplementary Figure 8. Ectopic expression of VNUT in dorsal horn neurons of *Slc17a9*$^{-/-}$ mice rescues tactile allodynia after PNI.
(a) Real-time PCR analysis of *Slc17a9* mRNA using total RNA extracted from neuronal cell line Neuro2A cells, transduced with pZac-NSE-AcGFP or pZac-NSE-VNUT vectors. Values represent the relative ratio of *Slc17a9* mRNA (normalized to the value for *18s* mRNA) to cells with pZac-NSE-AcGFP (*n=6*). **(b)** Measurement of $[ATP]_e$ content in the culture media of Neuro2A cells, transduced with pZac-NSE-AcGFP or pZac-NSE-VNUT, with or without treatment of Ca2+ ionopohre ionomycin (5 μM) for 20 min. Values represent the relative ratio of ATP levels to the control (*n=5*). **(c)** Real-time PCR analysis of *Slc17a9* mRNA using total RNA extracted from the spinal cord and the DRG of WT mice 28 days after intraspinal viral vector injection (AAV-NSE-AcGFP or AAV-NSE-VNUT). Values represent the relative ratio of *Slc17a9* mRNA (normalized to the value for *18s* mRNA) to the AAV-NSE-AcGFP mice (*n=6*). **(d)** PWT of *Slc17a9*$^{-/-}$ mice intraspinally injected with AAV-ESYN-AcGFP or AAV-ESYN-VNUT viral vectors before and after PNI (AAV-ESYN-AcGFP;*Slc17a9*$^{-/-}$: *n*=6, AAV-ESYN-VNUT;*Slc17a9*$^{-/-}$: *n=7*, *$*P<0.05$*, *$***P<0.001$* vs. AAV-ESYN-AcGFP;*Slc17a9*$^{-/-}$, two-way ANOVA with post hoc Bonferroni test). Values are means ± s.e.m.
